# Quality-of-Life Assessment and Pharmacokinetic Study in Hemophilia A Patients Undergoing Prophylactic Treatment

**DOI:** 10.3390/pharmacy13010016

**Published:** 2025-02-02

**Authors:** Nikolaos Kotsiou, Paschalis Evangelidis, Michail Bolios, Konstantinos Tragiannidis, Panagiotis Kalmoukos, Zacharo Ntova, Sofia Chissan, Sofia Vakalopoulou, Eleni Gavriilaki

**Affiliations:** 2nd Propedeutic Department of Internal Medicine, Hippokration General Hospital, 54642 Thessaloniki, Greece; pascevan@auth.gr (P.E.); boliosmike@gmail.com (M.B.); konstantinos.tragiannidis@gmail.com (K.T.); kalmoukosp@yahoo.gr (P.K.); ntovairo@gmail.com (Z.N.); sofiachissan@yahoo.com (S.C.); svakalopoulou@yahoo.com (S.V.); elenicelli@yahoo.gr (E.G.)

**Keywords:** hemophilia A, health-related quality of life, pharmacokinetic study, Greece, Haem-A-QoL, prophylactic treatment

## Abstract

This study evaluates the health-related quality of life (HRQoL) among patients with hemophilia A currently undergoing prophylactic treatment at the Hemophilia Center of Northern Greece. Using the Haem-A-QoL questionnaire, we assessed various HRQoL dimensions in a cohort of 29 adult male patients, analyzing the impact of age, disease severity, and treatment regimens. The results revealed that younger patients (18–30 years old) exhibited significantly better overall HRQoL scores (total score of 25.36) compared to older age groups (37.81 for the 31–45 group and 43.71 in the 45+ group), particularly in the physical health (29.16 vs. 48.43 vs. 58.57) and mental well-being domains (25 vs. 37.11 vs. 41.07). Interestingly, moderate hemophilia patients reported lower HRQoL (42.31) than those with severe form (34.85), suggesting unique challenges in managing their condition. The ’Sports/Free Time’ domain had the highest scores (65.81), indicating significant limitations in physical activities in the everyday lives of affected individuals. However, better outcomes were observed in the mental dimension (36.09), work/study (34.88), family planning (10.68), and relationships aspects (16.67), where our cohort reported very low scores compared to similar studies, indicating a significantly better quality of life in these domains. These findings highlight the importance of personalized psychosocial support and targeted interventions to address the specific needs of hemophilia patients, particularly in enhancing physical activity opportunities and managing the psychological burden of moderate hemophilia. The study contributes valuable insights into the HRQoL of hemophilia patients and underscores the necessity for tailored approaches to improve patient outcomes across all dimensions of life.

## 1. Introduction

Hemophilia is a hereditary genetic disorder characterized by frequent bleeding episodes. It is an X-linked recessive condition, predominantly affecting males, and arises due to the deficiency of coagulation factor VIII (hemophilia A) or factor IX (hemophilia B) [1,2]. Hemophilia A is a rare hematological disorder, affecting approximately 1 in 5000 individuals, while hemophilia B affects 1 in 30,000 [1]. The risk of bleeding is correlated with the severity of the condition, which is classified as mild (5–40%), moderate (1–5%), or severe (<1%) based on the factor levels in functional plasma [3]. In severe cases, hemophilia can become a significant clinical challenge; however, nowadays it typically reduces life expectancy by only a few years compared to the healthy population [4]. Recurrent bleeding episodes in joints and muscles are the most prevalent symptoms of the condition and may have a variety of long-term clinical repercussions, including chronic musculoskeletal disruptions, joint diseases, constant pain, and limited range of motion [5,6]. In addition, the regularity of treatment injections, the absence from school/work due to hospital visits, clinical manifestations, and limited engagement in social activities, such as sports, are typical [7]. Thus, hemophilia and its management have a significant effect on the health-related quality of life (HRQoL) of affected individuals. The World Health Organization’s (WHO) definition of HRQoL is ‘an individual’s perception of their position in life in the context of the culture and value systems in which they live and in relation to their goals, expectations, standards and concerns’ [8]. Based on this definition, the term includes a range of patients’ mental, emotional, physical, social and behavioral aspects [9]. In short, HRQoL is directly affected by parameters like disease severity and management strategies, the existence of comorbidities, personal socioeconomical status and living environment, and each individual’s coping mechanisms of pain, anger, and anxiety [10,11].

Advances in treatment have significantly improved life expectancy for hemophilia patients [4,12,13,14]. In mild and most moderate cases, the management strategy includes symptomatic treatment, involving the administration of clotting factors only during bleeding episodes or when there is a risk of hemorrhage, such as major surgeries (on demand treatment). In contrast, severe cases require continuous prophylactic treatment, necessitating frequent injections, hospital visits, and regular social absenteeism throughout the patient’s life [15,16]. This management strategy significantly impacts an individual’s daily life, social activities, and mental health [16,17,18]. Accurate HRQoL evaluation can inform treatment decisions, optimize therapeutic strategies, and enhance overall supportive care for hemophilia patients [19,20,21]. Self-assessment by patients is also vital for personalizing treatment, as it can reveal important subtle characteristics, such as anxiety or depression, that may not be readily apparent to healthcare providers [22,23,24].

Standard half-life factor VIII (SHL FVIII) products have been the cornerstone of hemophilia A treatment, providing a temporary elevation of FVIII levels to prevent or manage bleeding episodes [4]. These products typically require frequent infusions, due to their relatively short half-life, which generally ranges from 8 to 12 h. This frequent dosing schedule can place a substantial strain on patients, impacting their quality of life and adherence to treatment. In response to these challenges, extended half-life (EHL) FVIII products have been developed. EHL FVIII products are engineered to remain in the bloodstream longer, thereby extending the dosing intervals to every 3–7 days, depending on the specific product and individual patient pharmacokinetics. The advent of EHL FVIII products has significantly improved the management of hemophilia A, offering patients greater flexibility, reducing the frequency of infusions, and potentially enhancing adherence and overall quality of life [25].

Pharmacokinetic (PK) studies have emerged as a crucial tool in the personalized management of hemophilia, enabling healthcare providers to tailor prophylactic treatments to each patient’s specific needs [26]. Traditional prophylaxis regimens may lead to suboptimal dosing, either increasing the risk of bleeding or unnecessarily frequent infusions [27]. Population pharmacokinetics (PopPK), utilizing Bayesian models, allows for the development of individualized PK profiles that consider factors such as age, weight and factor concentrate type. These profiles can predict how long a patient will maintain therapeutic levels of clotting factors, thereby optimizing treatment schedules and reducing the overall burden of therapy [28].

The implementation of PK-tailored prophylaxis has shown promise in improving clinical outcomes for hemophilia patients by reducing the frequency of bleeds and enhancing the overall quality of life [26,27,28,29,30]. Tools like the Web-Accessible Population Pharmacokinetic Service—Hemophilia (WAPPS-Hemo ^©^) (McMaster University, Hamilton. Canada) facilitate the integration of individualized PK data into routine clinical practice, thus allowing clinicians to make informed decisions in alignment with the clinical and social needs of patients [31]. Despite the potential benefits, the widespread adoption of PK-tailored prophylaxis faces challenges, including patient recruitment and the models’ adjustments in newer therapies like emicizumab [26,28,32].

The primary objective of this study is to evaluate the HRQoL of hemophilia A patients undergoing prophylactic treatment at the Hemophilia Center of Northern Greece, utilizing the Haem-A-QoL questionnaire. Additionally, this research aims to examine the pharmacokinetic parameters of different FVIII products already included in their treatment regimens.

## 2. Materials and Methods

### 2.1. Study Population

The study included 29 consecutive male hemophilia patients registered and monitored at the Hemophilia Center of Northern Greece. Participants were classified based on factor levels: those with levels <1% were considered to have severe hemophilia, while levels 1–5% indicated a moderate form of the condition. The inclusion criteria required participants to be under prophylactic treatment, to be at least 18 years old with severe/moderate hemophilia, and capable of understanding the questions and willing to complete the questionnaire. The questionnaire and the blood sampling were conducted in person at the center between June 2023 and September 2023. Participation was voluntary, while parameters like age, height, weight, disease severity, baseline factor levels, blood type, the factor VIII product being used in prophylaxis, and dose regimen were collected. The study complied with the Declaration of Helsinki and was approved by the Institutional Review Board of Hippokration General Hospital, with informed consent collected from all participants. To ensure patient confidentiality, all collected data were anonymized, analyzed and stored securely, with access restricted to authorized personnel only. The approval code of the study was 585/25.7.2019.

### 2.2. Measuring Tools

For our study, the Haem-A-QoL^©^ (Haemo-QoL^©^ group, Hamburg, Germany) quality-of-life index questionnaire for adults with hemophilia was employed. This questionnaire was selected for our research because it has been extensively tested in various studies to ensure its reliability and specificity [11]. The questionnaire consists of 46 items distributed across 10 dimensions, including physical health, emotional well-being, self-perception, leisure and sports, work and school, condition management, treatment satisfaction, future outlook, family planning, and partnership-sexuality, as well as a cumulative score scale [33]. Scores are calculated by converting the raw scores for each dimension and the overall score to a scale of 0 to 100, where 0 indicates the best health-related quality of life and 100 reflects the worst [34]. In our study, we employed the Greek version of the Haem-A-QoL^©^ questionnaire, used with permission from the Haemo-QoL^©^ group [35].

FVIII levels were measured from plasma samples (one-stage clotting assay, Pathromtin SL reagent) taken before and 2 h after the administration of the factor concentrate, according to each patient’s normal prophylaxis regimen. Pharmacokinetic models were generated using the McMaster PopPK^©^ clinical calculator (Version 1.2 (2022-05-04)^©^ 2024 McMaster University, Hamilton, Canada), powered by WAPPS-HEMO^©^. The tool was utilized after the expressed permission of the McMaster group.

### 2.3. Statistical Analysis

Sample size was calculated based on estimated QoL differences between severe and moderate hemophilia. Power was defined at 80% for a 1% level of significance. Total sample size was determined at 25 subjects. The mean and standard deviation were utilized for the descriptive analysis of quantitative variables, while relative frequencies and absolute values (n, %) were applied to qualitative variables. The Shapiro–Wilk normality test was employed for variables with fewer than 50 subjects. To investigate correlations between the scores of the 10 subscales and the overall score of the Haem-A-QoL questionnaire in relation to factor concentrates, disease severity, and age groups, comparisons were conducted among three or more independent groups. When subscale scores followed a normal distribution, a parametric one-way analysis of variance (ANOVA) was applied; otherwise, when the assumption of normality was not met, a non-parametric Kruskal–Wallis test was used. Similarly, to assess correlations between the Haem-A-QoL subscale scores and the overall score with regard to prophylaxis, comparisons were performed between two independent groups. The parametric Independent Samples t-test was applied for normally distributed subscale scores, whereas the non-parametric Mann–Whitney test was utilized when normality was not observed. Non-parametric tests are robust to deviations from normality and do not rely on distributional assumptions; thus, they are suitable for analyzing non-normally distributed data. The χ^2^ test was utilized to examine associations between clinical and demographic characteristics. Data analysis was executed by using the statistical software “IBM SPSS Statistics, Version 23.0,” with the significance level established at 5%.

## 3. Results

For this research, a group of 29 adult males with hemophilia A completed the Haem-a-QoL questionnaire and agreed to blood sampling. Participants ranged in age from 19 to 70 years and exhibited severe and moderate hemophilia. The sample population was geographically diverse, including individuals from both rural and urban areas across Thrace, Macedonia, Thessaly, and the North Aegean islands. The detailed demographics of the participants are presented in Table 1.

A significant distribution was identified between age groups and disease severity. Specifically, a considerable proportion (44.8%) of patients with severe hemophilia were in the 31–45 age group, a percentage markedly higher than that observed in the 18–30 (20.6%) and 45+ (10.3%) age groups. In contrast, among those with moderate hemophilia, the distribution of patients was more consistent across the 18–30 and 45+ age groups, with percentages of 10.3% and 13.7%, respectively. The distribution between age groups and disease severity is available as Supplementary Material in the Word file entitled ‘Distributions’.

Table 2 displays the standard deviation, mean score, minimum, and maximum values for the scores from each of the 10 subscales of the questionnaire as well as the overall score, categorized by age group. Data indicate that the 18–30 individuals have a significantly lower mean score (25.36) compared to the other two age groups (37.81 and 43.71, respectively), suggesting a markedly better quality of life. In particular, the domains of family planning and relationships stand out with a notably low score of 4.16, reflecting a positive outcome. Similarly, low scores are observed in these domains for the other age groups (12.89–23.43 and 12.5–15.47), indicating an overall good quality of life in these areas. However, the highest score, and thus the lowest quality of life, is found in the sports/free time domain, which includes questions related to avoiding sports due to hemophilia, limitations on freedom to travel, and the necessity of planning activities in advance. Additionally, the only domain where the 18–30 age group exhibits a higher score, and thus a lower quality of life, is in coping with the disease. This domain includes questions about the patients’ efforts to recognize bleeding episodes promptly, understand whether they are bleeding, and manage the control of such episodes.

Table 3 encompasses the statistical parameters (mean value, standard deviation, and minimum and maximum values) across each of the 10 questionnaire subscales, categorized by condition severity, alongside the total score.

Although the study involved a small sample size, it is particularly noteworthy that both the overall questionnaire score and the scores on 9 of the 10 individual sub-scales were higher in patients with moderate hemophilia. The exception was the treatment domain, which includes questions related to bleeding episodes, where patients with moderate hemophilia exhibited a better quality of life, likely due to the lower annual bleeding rates typically observed in this group. Consistent with earlier findings, the family planning and relationship domains yielded low scores, while the highest scores were again found in the category related to sports and social activities, reflecting significant limitations for affected individuals.

The standard deviation, mean score, minimum, and maximum values for the scores from each of the 10 subscales of the questionnaire as well as the overall score, categorized by the factor concentrate, are available as Supplementary Material in the Word file entitled ‘Scores—sFactor Concentrate’. Due to the uneven distribution of patients across FVIII products, definitive conclusions cannot be drawn; however, the family planning and relationship domains consistently scored low, while the sports/free time and physical health domains showed particularly high scores on a case-by-case basis, indicating substantial challenges for patients with hemophilia in these aspects of their daily lives.

Table 4 presents the mean scores for each of the 10 questionnaire subscales for the entire sample of 29 patients. The overall score of 37.02 suggests a relatively good quality of life across the cohort, with notably low scores recorded in the coping (18.04), family planning (10.68), and relationships (16.67) domains.

The analysis of each question individually revealed significant statistical differences based on the patient’s treatment regimen in various aspects of the questionnaire. These differences were particularly evident in how hemophilia affects patients’ lives (*p* = 0.007), their concern about the condition worsening (*p* = 0.021), fear of potential complications (*p* = 0.001), discomfort associated with both the frequency and the process of injections (*p* = 0.012), ability to attend work or school despite the disease (*p* = 0.001), levels of anger and pain (*p* = 0.019), the time required to prepare for daily activities (*p* = 0.014), and the sense of social exclusion experienced (*p* = 0.045). Significant statistically differences were also observed in responses related to injection procedure (*p* = 0.014) and the impact on daily activities (*p* = 0.022), depending on the severity of the disease. Additionally, significant differences were noted in the responses concerning overall physical health (*p* = 0.015), participation in sports activities (*p* = 0.004), and treatment-related complications (*p* = 0.015), which varied according to the age of the patients (Table 5).

**Figure 1:**
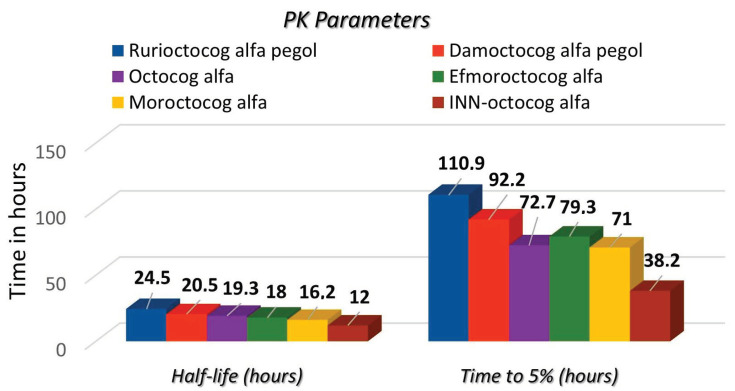
The pharmacokinetic properties of different FVIII concentrates used in the 29 patients’ prophylaxis regimens.

Concerning the pharmacokinetic properties of factor FVIII concentrates, rurioctocog alfa pegol demonstrated the longest half-life at 24.5 h, followed by damoctocog alfa pegol at 20.5 h, octocog alfa at 19.3 h, efmoroctocog alfa at 18 h, moroctocog alfa at 16.2 h, and INN-octocog alfa at 12 h. In terms of the average time required to reach 5% of FVIII levels, INN-octocog alfa had the shortest duration at 38.2 h, with moroctocog alfa at 71 h, octocog alfa at 72.7 h, efmoroctocog alfa at 79.3 h, damoctocog alfa pegol at 92.2 h, and rurioctocog alfa pegol at 110.9 h following (Figure 1).

## 4. Discussion

This study from the Hemophilia Center of Northern Greece evaluated the HRQoL of patients with hemophilia A undergoing prophylactic treatment, along with the pharmacokinetic properties of the factor VIII concentrates that are being used. By utilizing the Haem-A-QoL questionnaire, we assessed various dimensions of HRQoL, revealing significant differences based on age and disease severity.

Notably, our study found that patients aged 18–30 exhibited significantly better overall HRQoL scores compared to older age groups (25.36 vs. 37.81 vs. 43.71), particularly in the domains of physical health (29.16 vs. 48.42 vs. 58.57), mental well-being (25 vs. 37.11 vs. 41.07), and perception (25.83 vs. 38.75 vs. 47.14). This finding aligns with the results of our previous study, where the highest HRQoL scores were observed in the same age group, along with patients over 60 years old with mild hemophilia, while the lowest was in individuals aged 46–60 [36]. The early initiation of primary prophylactic therapy in younger patients, especially with extended half-life products, has been shown to mitigate the severity of hemophilic complications, thereby significantly enhancing their HRQoL. This observation aligns with the extant literature, which suggests that early prophylactic intervention in young patients with severe hemophilia is associated with improved HRQoL outcomes [37,38,39,40]. The beneficial effects of early prophylaxis were also highlighted in a non-interventional study which examined 94 patients from Asia, North America, Oceania, and Europe. In this study, individuals under prophylaxis treatment showed substantially improved total HRQoL compared with on-demand (26.6 vs. 40.1) [41]. In contrast, older individuals who either received insufficient treatment or were untreated during childhood and adolescence have developed severe difficulties [42]. This progression has markedly impaired their HRQoL, primarily due to mobility constraints, reduced physical activity, and diminished independence. These limitations, in conjunction with chronic pain, often lead to feelings of irritability, anger, and helplessness [43,44,45,46].

Another critical finding of our study was the impact of disease severity on HRQoL. While severe hemophilia generally correlates with poorer outcomes, due to the increased frequency of bleeding episodes and greater physical limitations, the moderate group in our study showed unexpectedly lower HRQoL in 9 out of 10 total domains, particularly in those related to mental health and coping with the disease, in disagreement with findings from other studies [47,48,49]. One hypothesis for this finding is that this cohort of individuals may not receive the same level of medical attention (no initiation of prophylaxis therapy) and psychosocial support as severe cases, potentially leaving them underserved [50,51]. Inconsistent bleeding patterns may also create uncertainty and stress, as these patients may not feel as adequately prepared to manage their condition.

To address these unique challenges, a multifaceted approach may be of utmost importance. For example, the implementation of comprehensive structured therapy, counseling programs, and potential patient education initiatives will offer psychosocial support and equip patients with treatment-related strategies for managing clinical symptoms [24,52,53,54,55]. Furthermore, to connect patients with others in similar situations and alleviate feelings such as social exclusion, depression, anxiety, and fear, peer support groups could be encouraged [56]. Lastly, given the fact that in the post-COVID era society has increasingly embraced remote methods for addressing various challenges, a shift towards the implementation of telehealth and digital applications that promote continuous monitoring, and guidance can be particularly valuable. Helpful paradigms are mobile health applications that empower patients by offering tools for symptom tracking, medication reminders, and providing educational content, thus fostering greater self-management and improving overall outcomes [57,58,59,60,61].

In terms of specific domains, we observed specific patterns across various dimensions. Specifically, our study revealed that the ’Sports/Free Time’ domain had the highest scores (a total score of 65.81), indicating the poorest quality of life. Participants in the study reported significant limitations not only in engaging in physical activities, a common issue among hemophilia patients due to the increased risk of bleeding and joint damage, but also impairment in traveling, due to need for exhaustive preparation. This result aligns with the findings of studies from India, Korea, Turkey, Brazil and Iran, where participation in physical activities and travel also emerges as one of the most impacted domains [62,63,64,65,66,67,68]. Our study also points out that the mental dimension (36.09), perception (38.39), family planning (10.68), and relationships (16.67) of the affected individuals showed better HRQoL outcomes, not only compared to other similar cohorts [69] but also compared with our previous study [36]. The increased HRQoL observed in these domains for our patients can be explained by the fact that our previous study included both on-demand and prophylaxis patients, but also due to the transition of the majority of patients under prophylaxis from SHL to EHL factor concentrates. The overall improvement compared with older studies highlights the advancements in medical management and psychosocial support that have transformed the field of hemophilia over the years [70].

This study provides a comprehensive evaluation of the HRQoL in hemophilia A patients undergoing prophylactic treatment and explores the pharmacokinetic properties of various factor VIII concentrates. Our findings reveal notable variations in HRQoL across age groups and disease severity, with younger patients demonstrating significantly better overall outcomes, particularly in physical health and mental well-being domains. Contrary to expectations, patients with moderate hemophilia reported lower HRQoL compared to those with severe forms, highlighting the possible unique psychosocial and management challenges faced by this subgroup. Our data underscore the substantial impact of hemophilia on patients’ everyday lives, particularly in domains like physical activity and social participation, as evidenced by high scores in the ‘Sports/Free Time’ domain. However, relatively lower scores in domains of family planning and relationships suggest better perceived outcomes in these areas among the study cohort compared to similar studies. From a pharmacokinetic perspective, given the small sample size of our cohort and due to the imbalanced proportion of individuals for different FVIII products, robust and valid conclusions about the role of factor concentrates cannot be drawn. These findings contribute to a growing body of evidence advocating for personalized therapeutic strategies that account for patient-specific factors such as age, severity, and psychosocial needs. Addressing gaps in physical activity opportunities and providing targeted psychological support could enhance the overall well-being of hemophilia patients.

## 5. Limitations and Future Work

The primary limitation of our research is the small sample size of patients whose data were analyzed. While these individuals represent the majority of patients receiving prophylactic treatment at our center, the limited sample size reduces the generalizability of our findings to other populations. Additionally, due to the imbalanced proportion of patients across different replacement products, safe and specific conclusions about the role of factor concentrates in HRQoL cannot be drawn. Moreover, the influence of coexisting conditions in affected individuals is not taken account in this study, since possible comorbidities were not established as an exclusion criterion. We also recognize the inherent limitations of self-reported data in HRQoL assessments, which rely heavily on each individual’s subjective perception. Furthermore, potential selection bias may exist, as patients who are more engaged in their treatment and motivated to participate in studies may report better HRQoL compared to those who decline participation. Such biases may have affected the accuracy and consistency of the results, particularly in domains like mental health and coping. Future research with larger and more diverse cohorts, longitudinal designs, and complementary assessment tools is essential to enhance the robustness and validity of these findings and address the limitations of this study.

## Figures and Tables

**Table 1:** The demographic characteristics of the sample population (*n* = 29).

Patients’ Data	Categories	Number of Patients (%)
**Age**	18–30 years	6 (20.7)
	31–45 years	16 (55.2)
	45+ years	7 (24.1)
**Height**	<170 cm	8 (27.6)
	171–180 cm	9 (31)
	180+ cm	12 (41.4)
**Weight**	<70 kg	5 (17.2)
	71–85 kg	17 (58.7)
	85+ kg	7 (24.1)
**Disease Severity**	Moderate	7 (24.2)
	Severe	22 (75.8)
**Blood Type**	0+	13 (44.8)
	0−	4 (13.7)
	A+	4 (13.7)
	A−	2 (6.8)
	B+	4 (13.7)
	B−	1 (3.4)
	AB+	1 (3.4)
**Factor Concentrates**	Rurioctocog alfa pegol	3 (10,3)
	Damoctocog alfa pegol	7 (24.1)
	Efmoroctocog alfa	15 (51.7)
	Octocog alfa	2 (6.8)
	Moroctocog alfa	1 (3.4)
	Inn-octocog alfa	1 (3.4)

**Table 2:** The statistical parameters of the 10 Haem-A-QoL questionnaire subscales, categorized by age group.

	Age Group					
	18–30		31–45		45+	
	*n*	Mean Score ± SD (Min–Max)	*n*	Mean Score ± SD (Min–Max)	*n*	Mean Score ± SD (Min–Max)
**Physical Health**	6	29.16 ± 11.33 (10–45)	16	48.43 ± 22.48 (15–95)	7	58.57 ± 19.11 (0–100)
**Mental Dimension**	6	25 ± 14.43 (0–43.75)	16	37.11 ± 18.94 (6.25–68.75)	7	41.07 ± 24.69 (0–100)
**Perception**	6	25.83 ± (16.93) (0–45)	16	38.75 ± 18.32 (5–65)	7	47.14 ± 20.15 (10–75)
**Sports/Free Time**	6	45.83 ± 25.06 (0–80)	16	72.5 ± 13.57 (35–90)	7	62.85 ± 29.74 (0–90)
**Work/Study**	6	23.95 ± 17.08 (0–50)	16	35.54 ± 14.61 (6.25–62.5)	7	38.39 ± 23.72 (0–62.5)
**Coping**	6	26.38 ± 11.2 (12.5–46.88)	16	17.71 ± 14.98 (0–50)	7	15.47 ± 16.32 (0–41.67)
**Treatment**	6	31.25 ± 11.12 (12.5–46.88)	16	37.11 ± 17.39 (9.38–75)	7	58.03 ± 18.43 (31.25–87.5)
**Future**	6	21.66 ± 12.13 (0–35)	16	35.62 ± 15.99 (5–55)	7	48.57 ± 20.43 (10–85)
**Family Planning**	6	4.16 ± 5.89 (0–12.5)	16	12.89 ± 20.42 (0–62.5)	7	12.5 ± 21.39 (0–62.5)
**Relationships**	6	4.16 ± 9.31 (0–25)	16	23.43 ± 26.71 (0–83.33)	7	15.47 ± 24.57 (0–58.33)
**Total Score** **Haem-A-QoL**	6	25.36 ± 8.96 (9.24–34.24)	16	37.81 ± 13.53 (15.22–64.13)	7	43.71 ± 19.51 (10.33–63.59)

**Table 3:** The statistical parameters of the 10 Haem-A-QoL questionnaire subscales, categorized by condition severity.

	Disease Severity			
	Moderate		Severe	
	*n*	Mean Score ± SD (Min–Max)	*n*	Mean Score ± SD (Min–Max)
**Physical Health**	7	53.57 ± 27.47 (0–100)	22	44.77 ± 23.32 (10–95)
**Mental Dimension**	7	43.75 ± 20.06 (0–100)	22	32.95 ± 20.65 (0–75)
**Perception**	7	45.71 ± 20.94 (10–75)	22	35.68 ± 18.84 (0–65)
**Sports/Free Time**	7	66.42 ± 21.47 (0–90)	22	64.09 ± 20.31 (0–85)
**Work/Study**	7	36.60 ± 23.01 (0–62.5)	22	32.95 ± 16.66 (0–62.5)
**Coping**	7	11.91 ± 10.78 (0–25)	22	21.21 ± 18.24 (0–66.67)
**Treatment**	7	52.23 ± 19.6 (31.25–87.5)	22	37.36 ± 17.76 (9.38–75)
**Future**	7	41.42 ± 21.49 (10–75)	22	34.09 ± 21.61 (0–85)
**Family Planning**	7	18.75 ± 23.14 (0–62.5)	22	8.52 ± 16.7 (0–62.5)
**Relationships**	7	20.23 ± 22.64 (0–58.33)	22	16.66 ± 25.37 (0–83.33)
**Total Score** **Haem-A-QoL**	7	42.31 ± 19.11 (10.33–63.59)	22	34.85 ± 14.08 (9.24–64.13)

**Table 4:** Mean scores for each of the 10 domains of the questionnaire of all the 29 patients.

Haem-A-QoL Domains	Score
**Physical Health**	48.71
**Mental Dimension**	36.09
**Perception**	38.39
**Sports/Free Time**	65.81
**Work/Study**	34.88
**Coping**	18.04
**Treatment**	40.73
**Future**	36.45
**Family Planning**	10.68
**Relationships**	16.67
**Total Score** **Haem-A-QoL**	37.02

**Table 5:** The quality-of-life dimensions that exhibited the most significant statistical variation, along with their corresponding varying factors.

Quality-of-Life Dimensions	Variable (*p*-Value)
**Patient’s Life Plans**	Factor concentrate (0.007)
**Concerns about Complications**	Factor concentrate (0.001)
**Potential for Deterioration**	Factor concentrate (0.021)
**Pain Levels**	Factor concentrate (0.013)
**Anger Levels**	Factor concentrate (0.019)
**Discomfort during Infusions**	Factor concentrate/Disease Severity (0.012)
**Disruption in Daily Activities**	Factor concentrate (0.001)
**Social Exclusion Perception**	Factor concentrate/Disease Severity (0.04)
**Total Physical Health**	Age of patients (0.015)
**Engagement in Physical Activities**	Age of patients (0.04)
**Treatment-related Complications**	Age of patients (0.04)

## Data Availability

The original contributions presented in the study are included in the article/Supplementary Material; further inquiries can be directed to the corresponding author.

## Supplementary Materials

The following supporting information can be downloaded at: https://www.mdpi.com/article/10.3390/pharmacy13010016/s1, Table S1: Distributions title; Table S2: Scores-Factor Concentrate.
