# Supplementary figures and images for: Quality-of-Life Assessment and Pharmacokinetic Study in Hemophilia A Patients Undergoing Prophylactic Treatment
Source: Pharmacy (Basel). 2025 Feb 2;13(1):16. doi: 10.3390/pharmacy13010016 (PMC11858962; doi:10.3390/pharmacy13010016)

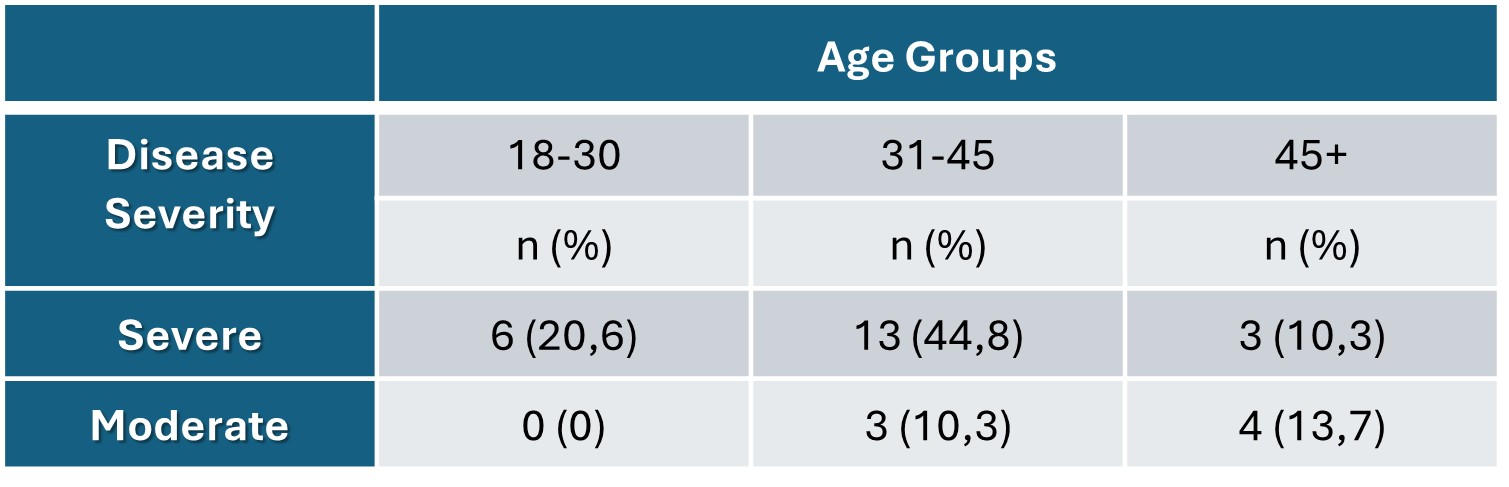

Supplement: Supplementary file 1 [file pharmacy-13-00016-s001.zip › Distributions (Supplementary Material).jpg]

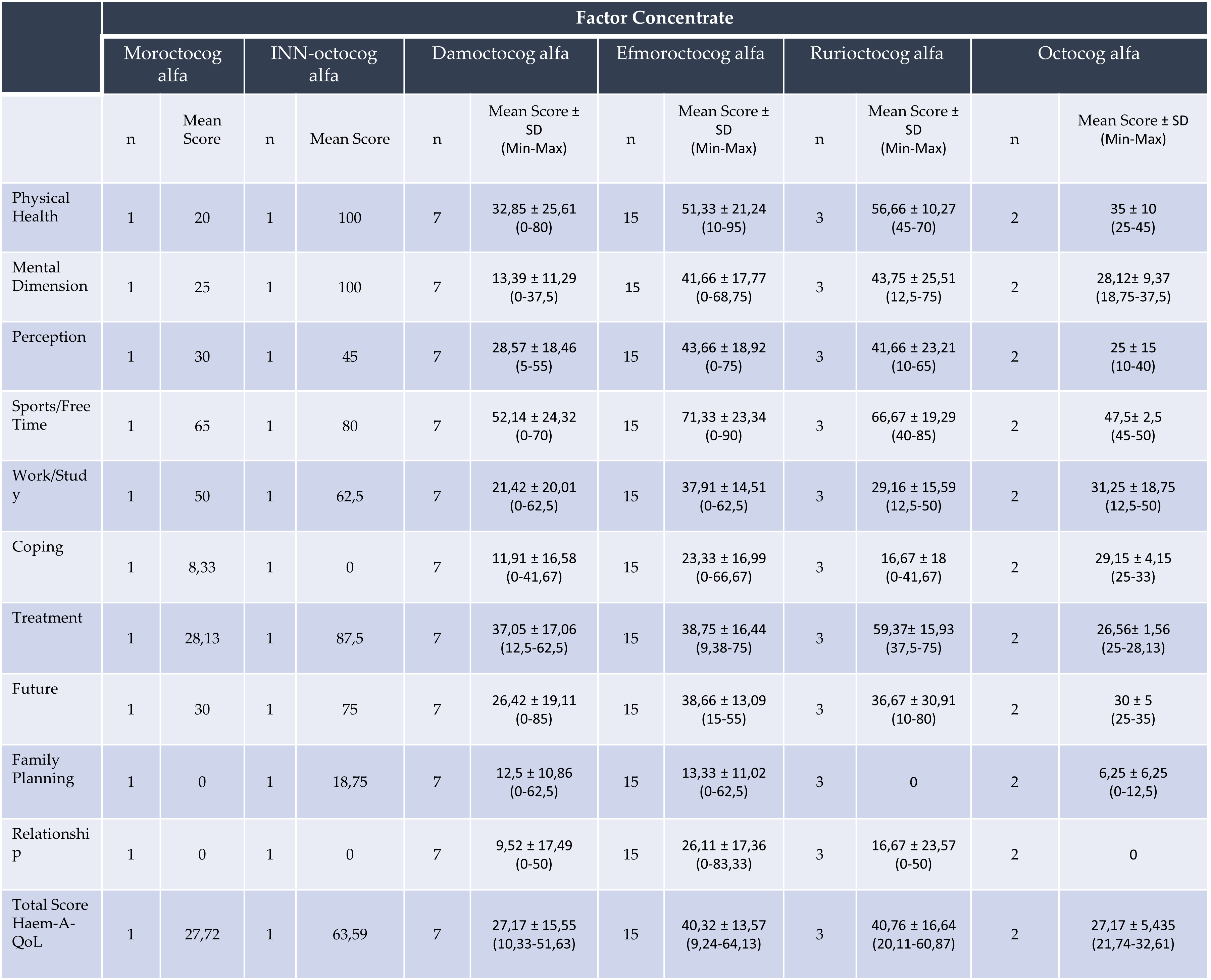

Supplement: Supplementary file 1 [file pharmacy-13-00016-s001.zip › Scores - Factor Concentrate (Supplementary).png]
